# Supplementary material for: A Comparative Study Between the Dietary Pattern in Argentina and International Recommendations
Source: Foods. 2025 May 28;14(11):1920. doi: 10.3390/foods14111920 (PMC12155276; doi:10.3390/foods14111920)
Supplement: Supplementary file 1 [file foods-14-01920-s001.zip › foods-3618249-supplementary.pdf]

Supplementary Materials

Table S1. Classification of Food Groups According to the categories established in EAT-LC

Food Group	Included Foods
**Red Meat**	Beef, pork, offal, processed meat
**Starchy Vegetables**	Fried potato, potato, sweet potato, corn
**Eggs**	Egg whites, egg yolks, whole eggs, fried eggs
**Poultry**	Chicken with crust, chicken without skin
**Dairy**	Skimmed milk, whole milk, grating cheese, grated cheese, soft cheese, semi-skimmed cheese, ricotta, whole yoghurt, non-fat yoghurt
**Fish**	Seafish, oily fish, fatty fish
**Vegetables**	Chard, chicory, garlic, artichoke, celery, fresh peas, aubergine, watercress, broccoli, squash, onion, cauliflower, string beans, asparagus, spinach, lettuce, cucumber, pepper, radish, cabbage, tomato, carrot, zucchini, pumpkin
**Fruit**	Pineapple, banana, plum, apricot, peach, strawberry, kiwi, lemon, tangerine, apple, melon, orange, butternut, grapefruit, grape
**Legumes**	Lentils, soybeans, peas, beans, chickpeas
**Grains**	Bread, cookies, crackers, pasta, flours, rice products, rye products, other cereals
**Nuts**	Almonds, peanuts, walnuts

Table S2. Classification of Food Groups Based on Food Balance Sheet (FBS) Data According to EAT-LC Recommendations

Food Group	**Included Foods**
Red Meat	Beef, lamb, goat meat, edible offal
Starchy Vegetables	Cassava and cassava-based products, potato and potato-based products, sweet potatoes
Eggs	Eggs
Poultry	Chicken meat
Dairy	Milk
Fish	Freshwater fish, demersal fish, pelagic fish, marine fish, crustaceans, cephalopods, molluscs, other aquatic animals
Vegetables	Onion, tomato, other vegetables
Fruits	Oranges, tangerines, lemons, limes, grapefruit, plantains, bananas, apples, pineapple, grapes, other fruits
Legumes	Beans, peas, chickpeas, lentils, soybeans, other legumes
Grains	Wheat and wheat-based products, milled equivalent rice, barley and barley-based products, corn and corn-based products, rye and rye-based products, oats, other cereals
Nuts	Almonds, peanuts, walnuts
